# Antioxidant Activity, Total Phenolic Content and Total Flavonoid Content in Sweet Chestnut (*Castanea sativa* Mill.) Cultivars Grown in Northwest Spain under Different Environmental Conditions

**DOI:** 10.3390/foods11213519

**Published:** 2022-11-04

**Authors:** Sidonia Martínez, Carlota Fuentes, Javier Carballo

**Affiliations:** Food Technology, Faculty of Science, University Campus As Lagoas s/n, University of Vigo, 32004 Ourense, Spain

**Keywords:** chestnuts, environmental conditions, total phenolics, total flavonoids, antioxidant capacity

## Abstract

The sweet chestnut fruit has always had great importance in the southern European countries. Chestnut production is an important source of income and a crop of high environmental value thanks to its role in soil protection. It is also a good food with enormous potential for various aspects of health because of its nutritional qualities. The quality of sweet chestnuts is affected by various factors, such as climatic conditions and cultivation inputs. It is very important to recognize the impacts of climate on chestnut fruits, to improve our current understanding of climate–chestnut interconnections. The current study investigated and compared the antioxidant activity and the total phenolic and flavonoid contents of different cultivars of chestnuts grown in different geographic areas of northwest Spain. The results obtained with three antioxidant capability assays (DPPH, ABTS and FRAP assays) were highly correlated. All the samples had high antioxidant capacity and high total phenolic and total flavonoid contents, which depended both on cultivar and growth region. Ventura variety, harvested in the coldest environments, presented the highest values of antioxidant activity (IC_50_^DPPH^ = 34.5 g/L), total phenolic content (131.84 mg equivalent of gallic acid/100 g FW) and total flavonoids (7.77 mg eq. catechin/100 g). The variations in the antioxidant capacity, total phenolic and total flavonoid contents of different cultivars, and their associations with climatic environmental factors, revealed the significant impacts of these factors on the synthesis of specialized metabolites and on the nutraceutical potential of chestnuts. The results can provide valuable information for selection of the cultivar and the cultivation conditions of the chestnut, in order to obtain chestnuts with high-quality bioactive characteristics.

## 1. Introduction

Chestnut fruits are food with highly significant economic, social and environmental importance. The genus *Castanea* Mill. is distributed in southern Europe, eastern North America, northern Africa, Asia Minor and eastern Asia [1]. *Castanea sativa*, commonly known as the European sweet chestnut, is the most consumed among the 12 chestnut species worldwide, and the predominant cultivated species in Europe. France, Italy and Spain have the main chestnut forests in Europe. *Castanea sativa* Mill is a very versatile plant because of its various useful properties and wide range of valuable products. Some varieties are marketed fresh and for candying (“marrons glacés”), whereas others produce nuts suitable for drying and flour production or are used for animal consumption [2]. Moreover, the chestnut contributes positively to the forestry landscape [3]. In some regions of Europe, mainly in the Mediterranean area, chestnuts are known as the “bread-tree,” because they provided basic food with nutritious and health-giving properties for people [4]. From the nutritional point of view, *C. sativa* fruits have high energy value, and they are good sources of starch, proteins, unsaturated fatty acids, free sugars, organic acids, vitamin C, vitamin E, minerals, carotenoids, phenolic compounds and alkaloids [5,6].

It is well known that the chemical composition of chestnuts can change due to a large number of factors. Different studies showed differences among species/cultivars [7,8] and among chestnuts from different geographic locations [8,9]. In addition, the seasonal variability and climatic conditions (temperature, sun exposure and rainfall), environmental conditions, cultivation techniques (soil, nutrients, minerals, irrigation, diseases and pests) and storage time influence their nutritional composition and quality [6,10].

In the Iberian Peninsula, chestnuts grow under a wide range of climatic conditions, from Mediterranean (with slight annual rainfall and a marked drought during the summer) to Atlantic (with higher precipitation per year) environments. However, the chestnut tree is very sensitive species to the summer drought, as this season is associated with high temperatures, and above all to the extension of the dry period. This limitation is especially important in the north of the Iberian Peninsula, where the average monthly rainfall is usually high, compared to the south of this peninsula, where rainfall is lower [11]. In Northern Spain, the chestnut groves that are cultivated today were selected mainly to provide food for a self-consumption society, so there is great varietal and intravarietal diversity [12]. As a consequence of this diversity, the harvested chestnuts come from varieties with highly variable nutritional and technological characteristics, and some of them are even not appropriate for industrial processing and/or not valued for fresh sale and are often used to feed livestock.

Nowadays, there is growing interest in the antioxidant properties of foods, and given the antioxidant and nutraceutical properties of chestnuts, there is great interest in obtaining more specific information on the different chestnut cultivars. Thus, more studies will be needed to further support their health benefits.

To determine the antioxidant capacity of foods, several methods that offers diverse and in most cases complementary information are available. These methods are based on the generation of free radicals that react with the sample in such a way that the antioxidant substances present in it produce a response by inhibiting these radicals.

This work aims to increase the knowledge about antioxidant potential and content of some bioactive components (total phenolic and total flavonoids) in different sweet chestnut cultivars of northwest Spain, in order to analyze the influences of variety and origin on these parameters. 

## 2. Materials and Methods

### 2.1. Samples

In this study, we analyzed 17 different samples of chestnuts (*Castanea sativa*) belonging to 9 cultivars from different geographical areas of northwest Spain: provinces of Ourense, Lugo and León (Table 1).

Each sample consisted of fruits from different representative trees of each cultivar and area, and three replicates (1 kg of fruit) of each sample were randomly collected. The chestnut fruits were harvested in October and November 2015 at the same phenological stage (cupula opening). The year 2015, for all the harvesting areas, could be described as a warm year. Regarding rainfall, 2015 was dry—precipitation was 26% below the value expected [13]. Climatic conditions are displayed in the Table 1. The data refer to the period between May and October 2015, a period that corresponds to the beginning of the vegetative period and maturation of the fruit, and were obtained from Meteogalicia (Meteorology Agency of Galicia, Spain) and AEMET (Meteorology Statal Agency of Spain).

After removing the outer and inner skins, nuts of each replicate from each sample were weighed, ground using a model AD5601 Moulinex (Italy) processor 800 W, mixed, vacuum packed and kept at −30 ± 2 °C until further use.

### 2.2. Chemicals

All chemicals and reagents were of analytical grade and were obtained from various commercial sources (Merck, Panreac, Scharlau and Sigma-Aldrich).

### 2.3. Moisture Determination

The moisture content was determined by drying in an oven at 105 ± 2 °C until obtaining a constant weight [14].

### 2.4. Preparation of Extracts

Three grams of ground nuts were homogenized in 20 mL of a solution of methanol:water (80:20, *v*/*v*). The samples were shaken for 15 min, sonicated for 15 min (Branson 3510 Ultrasonic Cleaner; Branson Ultrasonics Corporation, Danbury, CT, USA) and subsequently centrifuged for 10 min at 14,000 g in an Eppendorf centrifuge 5804R (Eppendorf AG, Ham-burg, Germany). The supernatant was collected, filtered through a 0.45 μm nylon filter and finally stored at −30 °C until analysis.

### 2.5. Determination of Antioxidant Activity

The antioxidant capacity was determined with three different methods: DPPH (2, 2-diphenyl-1-picrylhydrazyl) radical scavenging activity, FRAP (ferric reducing ability of plasma) and ABTS (2,2-azinobis-3-ethylbenzothiazoline-6-sulfonic acid) procedures.

#### 2.5.1. DPPH Assay

The DPPH assay was performed according to the method of Brand-Williams et al. [15] with some modifications. Different concentrations of extract (0.3 mL) were mixed with 0.5 mL of a DPPH solution in ethyl acetate (0.5 mM). The reaction mixture was kept in the dark for 60 min. When the DPPH radical reacts with an antioxidant compound, it is reduced and changes color. The color changes were read as absorbance at 517 nm in a Shimadzu UV-1800 spectrophotometer (Shimadzu Corp., Kyoto, Japan) against a control solution prepared by mixing pure ethyl acetate (2 mL) and the DPPH radical solution (0.5 mL).

The antioxidant activity is expressed in terms of IC_50_^DPPH^. The IC_50_^DPPH^ is defined as the extract concentration (mg/mL) required to decrease the initial DPPH concentration by 50%. This value was calculated by linear regression analysis of the dose–response curve, which was obtained by plotting the radical scavenging activity against extract concentration. To calculate the IC_50_^DPPH^, different percentages of inhibition were determined as follows:

% Inhibition = [A_control_−A_sample_)/A_control_] ∗ 100,

where A_control_ is the absorption of the blank at time 0 and A_sample_ is the absorption of the sample.

The Trolox equivalent of each extract (TRE) was calculated and is expressed as mmol Trolox equivalent (TRE) per 100 g of fresh matter (FM).

#### 2.5.2. Ferric-Reducing Antioxidant Power Assay (FRAP)

The FRAP was assessed according to Benzie and Strain [16], with some modifications. Briefly, 90 μL of extract solution was mixed with 250 μL of distilled water and 2.7 mL of working FRAP reagent prepared daily (10 mL of 0.3 M acetate buffer, pH = 3.6; 1 mL 10 mM TPTZ solution; and 1 mL of 20 mM FeCl_3_ 6H_2_O solution). The absorbance at 593 nm was recorded after 30 min of incubation at 37 °C in a Shimadzu UV-1800 spectrophotometer.

The antioxidant potential of samples was determined from a standard curve constructed using FeSO_4_•7H_2_O at a concentration range between 150 and 2000 μM. The results are expressed as μmol of Fe(II) per 100 g of fresh weight of sample (FM).

#### 2.5.3. ABTS Radical-Scavenging Activity

The antioxidant capacity using the ABTS method was determined according to Re et al. [17] with some modifications. ABTS (2,2′-azinobis-3-ethylbenzothiazoline-6-sulfonic acid) radical cation (ABTS^•+^) was produced by reacting an ABTS^•+^ solution (7 mM) with potassium persulfate (2.45 mM) for 16 h in the dark at room temperature. The ABTS^•+^ solution was diluted with ethanol to an absorbance of 0.70 at 734 nm. For the sample, 40 μL of extract solutions of chestnut was mixed with 4 mL of the ABTS^•+^ solution, and after 6 min in the dark at room temperature, the absorbance was read at 734 nm every 15 min for 90 min in a Shimadzu UV-1800 spectrophotometer.

Trolox was used as the standard. Results are expressed as mmol Trolox equivalent per 100 g of fresh matter (FM).

### 2.6. Determination of Total Phenolics

Total phenolic content was determined by the Folin–Ciocalteu colorimetric method [18]. Aliquots of 0.5 mL of extract were mixed with 2.5 mL of Folin–Ciocalteu reagent and 2 mL of NaCO_3_ (7.5 %; *w*/*v*). The mixture was heated at 45 °C for 15 min in a water bath in darkness and left to stand 30 min before the absorbance was measured at 765 nm. The total phenolic content is expressed as gallic acid equivalents (GAE)/100 g of fresh matter (FM) on the basis of a standard curve of gallic acid (0–100 mg/kg).

### 2.7. Determination of Total Flavonoids

Total flavonoid contents in the extracts were determined by the colorimetric method described by Zhishen et al. [19] with some modifications. The extract (1 mL) was mixed with 4 mL of distilled water and 0.3 mL of a 5% NaNO_2_ solution. After 5 min, 0.3 mL of a 10% AlCl_3_ H_2_O solution was added, and after 1 min, 2 mL of 1 M NaOH and 2.4 mL of distilled water were also added to prepare the mixture. The solution was mixed well, and the absorbance was read at 510 nm. (+)-Catechin (5–300 mg/kg) was used to construct the standard curves, and the results are expressed as mg of (+)-catechin equivalents per 100 g of fresh weight of sample.

### 2.8. Statistical Analysis

All analyses were carried out at least in triplicate. The data were examined by analysis of variance (ANOVA) with a confidence interval of 95% (*p* < 0.05), and the least squares difference test (LSD) was used to compare the mean values. The tests were implemented using Statistica software version 8.0 (Statsoft © Inc., Tulsa, OK, USA).

## 3. Results and Discussion

The moisture contents of chestnut fruits were between 48.4% (P3) and 63.4% (L1) (Table 2).

The results indicate a considerable variation of moisture—a significant difference between chestnut samples. Longal L1 had the highest moisture content. Longal L3 and Parede P3 had the lowest values. The values are similar to those described by Neri et al. [20], Otles and Selek [21] and Vasconcelos et al. [6] for different chestnut varieties.

Measurement of all of the individual antioxidant compounds in foods is very difficult because of the presence of numerous compounds with antioxidant potential. Several methods of estimating the total antioxidant activity in vegetables have been developed. In the present study, the antioxidant capacity of the different chestnut varieties was determined using three methods: ABTS, FRAP and DPPH assays. Antioxidant activities measured in methanol extract obtained through DPPH, FRAP and ABTS assays from a single extract were measured three times to test the reproducibility of the assays.

Table 2 shows the antioxidant capacity values obtained using the three methods under study and the IC_50_^DPPH^ values (50% inhibitory concentration of DPPH˙) in the different varieties of chestnuts analyzed. Usually, chestnuts have a high antioxidant capacity when compared to other foods. A good antioxidant capacity was found for all chestnut varieties studied in the present work. However, DPPH, FRAP and ABTS assays exhibited a high variation in antioxidant capacity among varieties. The DPPH values of chestnut samples under study ranged from 49.26 (P3) to 219.69 (V1) meq. Trolox/100 g of fresh sample. The total antioxidant capacity determined through the ABTS assay ranged from 74.0 (F1) to 371.8 (V1) meq. Trolox/100 g. The FRAP assay also showed a wide variation from 607.5 (P3) to 2899.2 (V1) μM Fe(II)/100 g. High antioxidant capacities were found in the same set of varieties in the three assays. Ventura variety, and specifically sample V1, presented the highest values of antioxidant activity for all three methods. This variety also had the lowest values of IC_50_^DPPH^ (34.5 g/L), which corroborates its high antioxidant capacity. The lowest antioxidant capacity was observed in Parede 3 (P3); it had significantly higher IC_50_^DPPH^ values reaching 146.86 g/L.

In previous studies described in the literature, the antioxidant activity of different chestnut varieties has been measured by several different methods, and it is difficult to compare the results. Neri et al. [20] found values from 302 to 311 mmol Trolox equivalents/100 g, using an ABTS^•+^ radical cation decolourization assay, in three Italian chestnut varieties. Blomhoff et al. [22], also for Italian chestnuts, reported 755 μmol per 100 g by using the FRAP antioxidant activity method.

**Figure 1:**
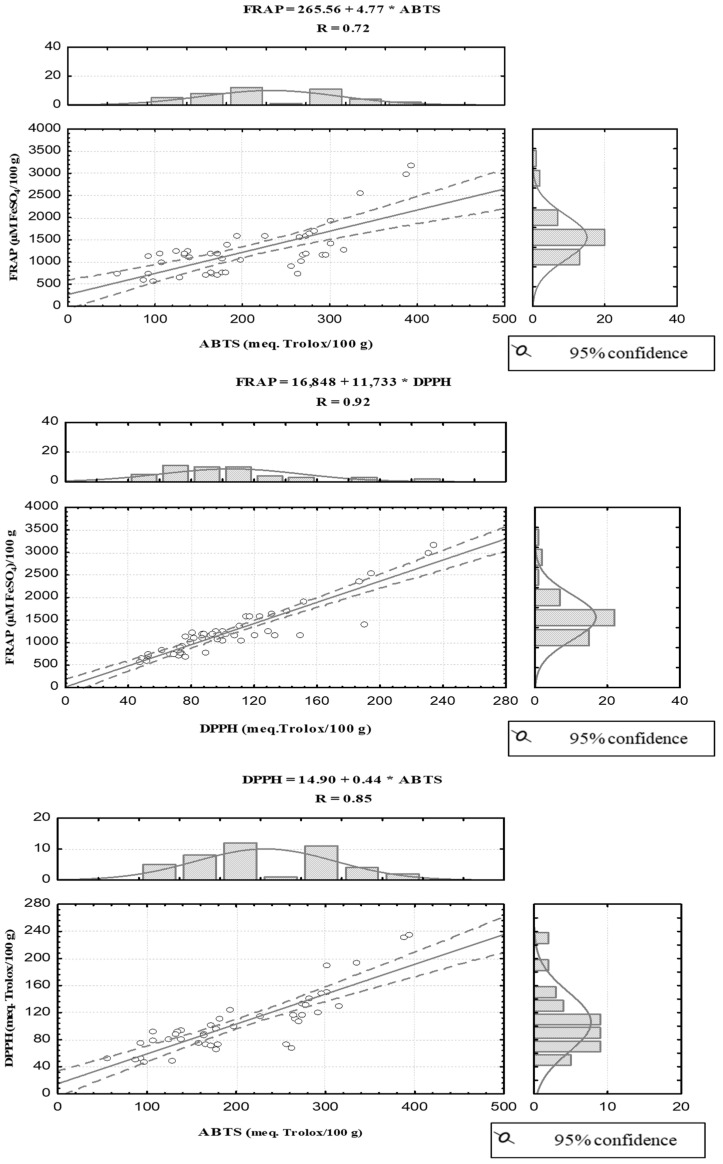
Correlations established between the results of the three antioxidant assays in the chestnut samples.

The correlation coefficients (r) between the three antioxidant assays were established and are presented in Figure 1. 

The highest correlation coefficient (r) was determined to be 0.92 between DPPH˙ and FRAP values, followed by 0.85 between DPPH˙ and ABTS^•+^. The lowest correlation coefficient (r) was between FRAP and ABTS^•+^ (r = 0.72). Our result agrees with previous studies that reported high correlations between different methods used for determining antioxidant activity [23,24].

There were significant differences between the antioxidant activity levels of the varieties, and within the same variety, between chestnuts harvested in different areas. Pearson correlation analyses were conducted to define the correlative relationships between antioxidant activities, total phenolic content and total flavonoids and environmental parameters (Table 3).

Antioxidant capacity was positively correlated with altitude, rainfall and average sunlight duration, and negatively correlated with maximum temperature. These results show that antioxidant capacity depends on geographical and climatic factors and not only on the chestnut variety. Environmental factors condition the physiological and reproductive cycles of species, and cause changes in their yields and fruit-quality characteristics. Increased concentrations of specialized metabolites is one of the survival strategies against the effects of adverse environmental factors [25]. Environmental differences contribute to the differences in antioxidant activity of chestnuts of the same variety. In this study, the coldest environments seemed to increase the antioxidant activity. The Ventura variety is frequently found in eastern areas of Galicia, at altitudes between 800 and 1000 m. These altitudes are higher than those of other chestnut varieties, so it is to be assumed that the average temperature is also lower. Altitude is an overall reflection of many ecological factors, such as temperature, humidity and sunlight duration. The increase in UV radiation in areas with higher altitudes could also influence the higher antioxidant capacities of some of the chestnut varieties, such as the Venture variety. The Parede variety, sample 3, was collected in Ponferrada (León, Spain), where the climate is warm and there is a summer drought characteristically, which may condition the characteristics of these chestnuts. Dinis et al. [23] pointed out the relationship between chestnut antioxidant activity and the edaphoclimatic conditions. These authors also reported that antioxidant capacity showed higher values in chestnuts (cultivar “Judia”) harvested in colder climates than in warmer environments. Figueroa et al. [26] reported that low temperatures (mainly when it is close to 0 °C), low humidity and an increase in the annual rainfall increase the antioxidant capacity of walnuts. These factors can affect metabolism and specialized metabolite accumulation.

The relationships between environmental conditions and varieties also affect the phytochemical compounds of fruits [27]. Neri et al. [20] reported that different clones of the same variety might exhibit different chemical compositions. Furthermore, variation due to harvesting year and the interaction between year and cultivar can also be important. According to a study by Dinis et al. [28], the morphological and phenological differences among different ecotypes of the Portuguese chestnut (Judia cultivar) are related to the small genetic variations and phenotypic adaptations to different climatic conditions. Fischer et al. [29] pointed out that an increases in altitude cause an increase in antioxidant content in various fruit species, a reaction to increasing UV light. It is known that various factors, such as the climatic conditions, cultivars, geographical area, soil nutrients and water availability may affect the bioactive composition of chestnut fruits. Barros et al. [30] found TEAC (Trolox equivalent antioxidant capacity) values between 0.564 and 1.046 mmol Trolox/kg in various chestnut varieties, and they pointed out that the antioxidant capacity depends on the crop.

Total phenolics are the main group of specialized plant metabolites in chestnuts. The total phenolic and flavonoid contents of the chestnut samples are also presented in Table 2.

Total phenolic contents ranged between 47.48 and 131.84 mg GAE/100 g FW. Ventura variety, sample V1 specifically, had the highest total phenolic content, followed by Longal (L3 and L4) (96.59 and 85.97 mg GAE/100 g, respectively), Xudía variety (92.52 mg GAE/100 g) and Ventura 2 (V2) (83.58 mg GAE/100 g). The lowest values were obtained for the Longal (L1 and L2) (62.98 and 47.48 mg GAE/100 g, respectively) and Parede (P1, P2 and P3) varieties (55.96, 62.68 and 50.69 mg GAE/100 g, respectively).

Different results have been reported in various studies. Hernández-Suárez et al. [31] found high total phenolic content (124 ± 29 mg/100 g) in different varieties of chestnuts from Tenerife (Spain). Echegaray et al. [32] found mean values of 130.00 mg GAE/100 g FW in a mix of four Spanish chestnut cultivars (Amarelante, Famosa, Longal and Judía). Kalogeropoulos et al. [33] observed total phenolic content of 43.0 ± 2.1 mg GAE/100 g of fresh weight in chestnuts from Greece. Abe et al. [34] found total phenolic content in Brazilian chestnuts of 92 mg/100 g. Nazzaro et al. [35] reported for Italian cultivar “Palomina” a mean value of 76.3 mg GAE/100 g. Chang et al. [36] studied five different Chinese chestnuts (*C. mollissima* Blume), and they showed that the total phenolic content ranged from 42.8 to 58.6 mg GAE/100 g fresh weight.

There were significant differences between and within each variety, depending on the origin. Total phenolic content was also positively correlated with altitude, rainfall and average sunlight duration, and negatively correlated with maximum temperature (Table 3). Therefore, lower maximum temperatures, higher altitudes, more rainfall and greater sunlight increase phenolic content in chestnuts. Consequently, it seems that both the variety and the environmental conditions significantly condition the synthesis of these bioactive compounds in chestnuts. Ciucure et al. [37] reported that the climatic conditions play a significant role in the synthesis of bioactive compounds in chestnuts.

It is well known that phenolics have an important role in plant metabolism. They are accumulated in plants as a response to stressful conditions, such as harmful environmental circumstances (drought, extreme temperatures, pollution, etc.) [38]. Xu et al. [24] studied the phenolic profiles of 21 chestnut samples collected from six Chinese geographical areas. They observed significant variation in the phenolic profiles of the chestnut samples from the various Chinese regions. In this case, high average temperatures during the chestnut growing season increased phenolic content and antioxidant activity. This could be due to the high temperatures increasing the photosynthetic capacity of plants and therefore improving the production of specialized metabolites such as phenolic acids [39]. However, Dinis et al. [23] reported that total phenolic content had higher values in chestnuts harvested in colder climates than in warmer environments.

**Figure 2:**
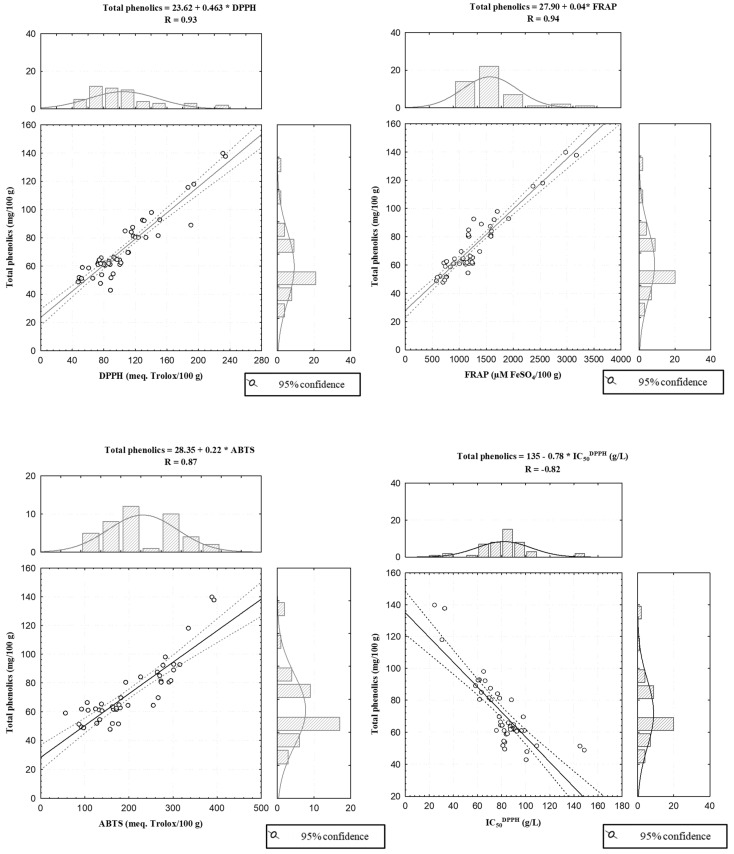
Correlations established among total phenolic content, results of the antioxidant assays and IC_50_^DPPH^ values of the chestnut samples.

We observed a high positive correlation between the content of total phenols and the antioxidant capacity obtained with the three antioxidant assays (Figure 2), suggesting that the predominant source of antioxidant activity could be the phenolic compounds. However, in the case of chestnuts, their antioxidant activity is only partially based on phenolic compounds, since the high content of ascorbic acid in chestnuts has an important contribution to antioxidant activity [40].

The highest correlation value was 0.94 between total phenolic content and FRAP (r = 0.94), followed by the correlation between total phenolic content and DPPH (r = 0.93). A high and negative correlation was found between total phenolic content and IC_50_^DPPH^ (r = −0.82). These results are in agreement with those reported by Barreira et al. [41], Chang et al. [36] and Xu et al. [24].

Flavonoids have different biological activities in plants, animals and bacteria. Flavonoid contents also varied widely in the present study between varieties and samples collected from different geographical areas, ranging from 2.20 to 17.77 mg eq. catechin (CAE)/100 g of FW. The largest amount was found in Ventura 1 (V1), followed by Longal 4 (L4) (11.99 mg CAE/100 g), Ventura 2 (V2) (11.59 mg CAE/100 g) and Rapada (R1) (11.09 mg CAE/100 g) samples. Echegaray et al. [32] found similar amounts of total flavonoids (8.58 mg CAE/100 g FW) in a mix of Spanish chestnuts. However, Dinis et al. [23] reported higher values (between 480 and 6720 mg CAE/100 g DW). They observed a significant correlation between total phenolic and flavonoid contents and antioxidant activity.

The highest total flavonoid content was observed in chestnuts from areas with higher precipitation, more sunlight hours and lower temperatures. The differences observed between samples harvested in different areas indicate that climatic conditions play an essential role in the synthesis of these phytochemicals in chestnuts. Total flavonoid content exhibited high positive correlations with altitude, rainfall and average sunlight duration. However, maximum temperature showed a negative correlation with total flavonoid content (Table 3). Strong sunlight and higher rainfall seem to promote the synthesis of more flavonoids in chestnuts. Zoratti et al. [42] reported than light is one of the most important environmental parameters affecting flavonoid biosynthesis in plants.

**Figure 3:**
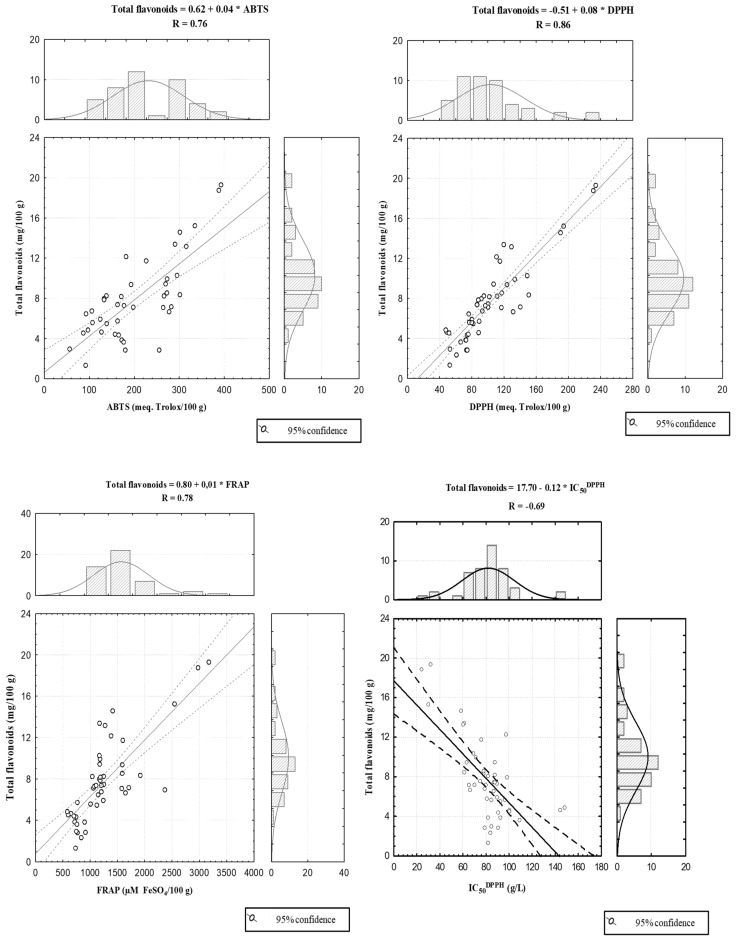
Correlations established between total flavonoid content, results of the antioxidant assays and IC_50_^DPPH^ values.

The correlation of total flavonoid content with antioxidant capacity is shown in Figure 3. There was a direct relationship between flavonoid content and total antioxidant activity. Pearson’s correlation coefficient analysis showed the presence of a significant positive correlation between total flavonoid content and DPPH assay results (r = 0.86). There were also high correlations between total flavonoids and ABTS and FRAP assay values (0.76 and 0.78, respectively). A negative correlation between total flavonoid content and IC_50_^DPPH^ (−0.69) was found. There was a higher correlation between antioxidant capacity and total phenolic content, indicating that other phenols, in addition to flavonoids, are available for inhibition of free radicals. However, the contributions of different bioactive compounds to total antioxidant activity can be difficult to know due to the synergistic effect and interactions among them [43].

A strong positive correlation (0.79) between the total phenolic content and total flavonoid content indicates that the flavonoids are the major polyphenols in chestnuts.

The flavonoids are associated with fragrance and taste of the fruit, and they have health benefits for humans associated with their bioactive properties, such as anticancer, anti-aging, anti-inflammatory, antibacterial or antiviral properties [44].

## 4. Conclusions

The results of the current study show that chestnuts have a high antioxidant capacity. Chestnuts contain significant concentrations of phenolics and flavonoids that are known to have positive effects in human health. Among the cultivars of chestnut from NW Spain we studied, Ventura 1 presented higher mean values for antioxidant capacity, total phenolic content and total flavonoids. High variability was observed among the geographical areas and cultivars of chestnuts. In this study, the cold seemed to increase the antioxidant activity, probably due to more thermal stress.

Based on these results, it could be concluded that differences among antioxidant activities and phenolic and flavonoids contents of chestnuts significantly depend on the variety but also on the environmental factors. It is relevant to consider the cultivar, but also cultivation location and environmental conditions in order to obtain the optimum content of desired bioactive compounds.

## Figures and Tables

**Table 1:** Information of chestnut samples used in the study and of the geographical areas. It was collected in northwest Spain (between May and October 2015, a period that corresponds to the beginning of the vegetative period and maturation of the fruit). Data were obtained from Meteogalicia (Meteorology Agency of Galicia) and AEMET (Meteorology Statal Agency).

Common Name	Codes	Geographical Area	Coordinates		Altitude (m)	Average Temp. (°C)	Minimum Temp. (°C)	Maximum Temp. (°C)	Rainfall (L/m^2^)	Average Sunlight Duration (h/month)	Commercial Features
			*Latitude*	*Longitude*							
Longal	L1	Parada do Sil, Ourense	42.38° N	−7.57° W	662–672	15.71	−1.5	39.9	44.03	210.0	Small caliber, between 95 and 100 chestnuts/kg
	L2	Monterroso, Lugo	42.79° N	−7.83° W	500–550	14.08	−0.2	37.3	59.17	198.1	
	L3	Castrelo do Val, Ourense	41.99° N	−7.42° W	417–570	16.57	−1.6	38.7	36.68	246.3	
	L4	A Gudiña, Ourense	42.06° N	−7,14° W	850–981	14.75	−1.9	34.2	92.43	259.3	
Famosa	F1	Monterroso, Lugo	42.79° N	−7.83° W	500–550	14.08	−0.2	37.3	59.17	198.1	Medium caliber, between 70 and 85 chestnuts/kg
	F2	Rubiá, Ourense	42.47° N	−6.89° W	420–577	16.95	−0.8	38.3	53.36	254.7	
	F3	Castrelo do Val, Ourense	41.99° N	−7.42° W	417–570	16.57	−1.6	38.7	36.68	246.3	
Parede	P1	Noceda del Bierzo, León	42.71° N	−6.40° W	750–838	11.20	−4.8	33.6	41.20	254.6	Small caliber, more than 110 chestnuts per kg
	P2	Monterroso, Lugo	42.79° N	−7.83° W	500–550	14.08	−0.2	37.3	59.17	198.1	
	P3	Ponferrada, León	42.55° N	−6.60° W	522–750	19.00	−1.0	38.7	40.00	254.6	
Ventura	V1	A Gudiña, Ourense	42.06° N	−7.14° W	850–981	14.75	−1.9	34.0	92.43	259.3	Small caliber, about 100 chestnuts/kg
	V2	A Mezquita, Ourense	42.24° N	−7.87° W	850–997	15.00	−1.9	34.4	91.30	259.2	
Amarelante	A1	Vilariño de Conso, Ourense	42.17° N	−7.18° W	726–950	15.17	−2.0	35.2	121.5	240.9	Large to medium size, between 55 and 90 chestnuts/kg
Raigona	RAI1	Rubiá, Ourense	42.47° N	−6.89° W	420–577	16.95	−0.8	38.3	53.36	254.7	Large to medium size, between 50 and 90 chestnuts/kg
Rapada	R1	A Gudiña, Ourense	42.06° N	−7.14° W	850–981	14.75	−1.9	34.0	92.43	259.3	Medium caliber, between 70 and 85 chestnuts/kg
Xudía	X1	Castrelo do Val, Ourense	41.99° N	−7.42° W	417–570	16.57	−1.6	38.7	36.68	246.3	Medium caliber, about 75 chestnuts/kg
Vilamaesa	VIL1	Castrelo do Val, Ourense	41.99° N	−7.42° W	417–570	16.57	−1.6	38.7	36.68	246.3	Medium caliber, about 75 chestnuts/kg

**Table 2:** Moisture content, antioxidant activities, total phenolic content and total flavonoids of the different chestnut cultivars.

Samples	Moisture Content (%)	DPPH (meq. Trolox/100 g)	ABTS^•+^ (meq. Trolox/100 g)	FRAP (μM Fe(II)/100 g)	IC_50_^DPPH^ (g/L)	Total Phenolics (mg eq. Gallic Acid/100 g)	Total Favonoids (mg eq. Catechin/100 g)
L1	63.4 ± 1.5 _b_	74.41 ± 2.2 _bc_	168.5 ± 4.7 _cd_	728.2 ± 17.8 _a_	89.52 ± 3.6 _d_	62.98 ± 2.50 _bc_	4.60 ± 0.91 _bc_
L2	53.4 ± 0.5 _ac_	84.51 ± 7.6 _bc_	160.0 ± 3.8 _cd_	733.2 ± 46.1 _a_	94.19 ± 11.3 _d_	47.48 ± 4.41 _a_	4.91 ± 0.71 _bcd_
L3	47.1 ± 0.2 _g_	151.65 ± 34.8 _fg_	287.2 ± 20.4 _fe_	1958.4 ± 392.5 _e_	70.00 ± 12.0 _bc_	96.59 ± 17.55 _f_	7.94 ± 0.88 _e_
L4	55.4 ± 0.3 _af_	118.68 ± 11.1 _de_	291.9 ± 5.4 _f_	1206.5 ± 56.9 _c_	61.80 ± 1.4 _b_	85.97 ± 6.14 _def_	11.99 ± 2.22 _f_
F1	58.6 ± 0.1 _e_	55.47 ± 4.7 _a_	74.0 ± 25.3 _a_	770.9 ± 58.7 _a_	83.70 ± 3.0 _cd_	55.71 ± 5.43 _abc_	2.20 ± 0.82 _a_
F2	54.2 ± 0.2 _acf_	99.35 ± 12.4 _cd_	208.9 ± 52.6 _e_	1100.8 ± 90.5 _bc_	81.84 ± 4.7 _cd_	65.81 ± 3.37 _c_	7.58 ± 0.58 _e_
F3	55.4 ± 0.3 _af_	95.52 ± 6.9 _cd_	160.5 ± 24.2 _cd_	1149.2 ± 64.26 _c_	92.46 ± 5.6 _d_	62.71 ± 1.82 _bc_	7.77 ± 0.45 _e_
P1	51.7 ± 0.3 _cd_	72.18 ± 2.8 _b_	178.3 ± 2.7 _e_	762.4 ± 6.6 _a_	94.24 ± 13.6 _d_	55.96 ± 5.86 _abc_	3.22 ± 0.56 _ab_
P2	51.1 ± 0.2 _d_	71.89 ± 2.9 _b_	259.0 ± 5.4 _f_	850.1 ± 95.9 _ab_	85.14 ± 7.9 _cd_	62.68 ± 2.37 _bc_	3.33 ± 0.70 _ab_
P3	48.4 ± 0.8 _g_	49.26 ± 2.1 _a_	103.7 ± 21.0 _ab_	607.5 ± 36.6 _a_	146.87 ± 2.6 _e_	50.69 ± 1.65 _ab_	4.85 ± 0.33 _bcd_
V1	55.8 ± 0.1 _fh_	219.69 ± 22.2 _h_	371.8 ± 32.2 _g_	2899.2 ± 320.0 _f_	34.50 ± 3.5 _a_	131.84 ± 12.04 _g_	17.77 ± 2.21 _g_
V2	54.4 ± 0.4 _acf_	157.52 ± 29.5 _g_	289.8 ± 14.8 _f_	1254.1 ± 137.3 _c_	63.98 ± 7.8 _b_	83.58 ± 4.69 _de_	11.59 ± 2.59 _f_
A1	57.4 ± 0.4 _eh_	95.73 ± 4.22 _cd_	135.6 ± 3.7 _bc_	1221.0 ± 47.6 _c_	78.74 ± 4.3 _c_	60.27 ± 5.43 _bc_	7.90 ± 0.40 _e_
RAI1	52.6 ± 0.1 _cd_	82.77 ± 3.2 _bc_	131.6 ± 10.2 _bc_	1156.1 ± 74.3 _c_	90.19 ± 2.2 _d_	61.46 ± 0.75 _bc_	6.25 ± 0.99 _cde_
R1	54.8 ± 0.1 _af_	116.40 ± 6.7 _de_	200.3 ± 23.3 _de_	1522.1 ± 122.4 _d_	82.38 ± 8.2 _d_	77.98 ± 7.49 _d_	11.09 ± 1.49 _f_
X1	53.4 ± 0.1 _ac_	129.42 ± 11.9 _ef_	274.5 ± 9.3 _f_	1644.1 ± 65.8 _d_	67.37 ± 2.9 _b_	92.52 ± 5.27 _ef_	6.97 ± 0.27 _de_
VIL1	53.4 ± 0.2 _ac_	82.94 ± 8.8 _bc_	101.8 ± 8.1 _ab_	1117.7 ± 101.7 _bc_	87.63 ± 8.7 _cd_	63.01 ± 2.90 _bc_	6.26 ± 0.61 _cde_

Values all represent the mean ± standard deviation of three replicates for each sample (*n* = 3). Mean values in the same column not followed by a common letter were significantly different (*p* < 0.05).

**Table 3:** Pearson correlation coefficients for antioxidant capacity by the DPPH, ABTS and FRAP assays, total phenolic content, total flavonoids and environmental conditions.

	Altitude (m)	Average Temp. (°C)	Minimum Temp. (°C)	Maximum Temp. (°C)	Rainfall (L/m^2^)	Average Sunlight Duration (h/month)
DPPH	0.53 *	−0.10	−0.20	−0.48 *	0.50 *	0.46 *
ABTS	0.50 *	−0.20	−0.24	−0.48 *	0.44 *	0.39 *
FRAP	0.37 *	−0.02	−0.15	−0.36 *	0.37 *	0.44 *
Total phenolics	0.48 *	−0.09	−0.20	−0.43 *	0.42 *	0.44 *
Total flavonoids	0.68 *	−0.05	−0.19	−0.58 *	0.65 *	0.59 *

* Marked correlations are significant at *p* < 0.05.

## Data Availability

Data are contained within the article.
